# Complete Genome Sequences of Pseudomonas alkylphenolica Neo and *Variovorax* sp. Strain CSUSB, Obtained in Undergraduate Microbiology Courses Using a Hybrid Assembly Approach

**DOI:** 10.1128/MRA.01520-19

**Published:** 2020-02-27

**Authors:** Christopher Ne Ville, Dylan Enright, Ivan Hernandez, Jeremey Dodsworth, Paul Orwin

**Affiliations:** a Department of Biology, California State University San Bernardino, San Bernardino, California, USA; University of Arizona

## Abstract

Two Gram-negative bacteria with a high G+C content were isolated from soil in undergraduate microbiology classes by enriching for low nutrient growth and neonicotinoid pesticide tolerance. DNA from these isolates was purified and sequenced using a hybrid approach. Here we report the genome sequences of Pseudomonas alkylphenolica strain Neo and *Variovorax* sp. strain CSUSB.

## ANNOUNCEMENT

Two Gram-negative bacteria were isolated from environmental soil samples by undergraduates in microbiology courses. Pseudomonas alkylphenolica strain Neo was initially isolated from garden soil using discs impregnated with the imidacloprid-containing pesticide Bayer Advanced fruit, citrus, and vegetable insect control (0.235% imidacloprid; ∼9.2 mM). Colonies growing closest to the discs were subsequently tested for growth in the presence of higher concentrations of the pesticide, and the isolate sequenced was selected based on growth in a 40% (vol/vol) pesticide challenge (∼3.67 mM imidacloprid). Initial characterization of *P. alkylphenolica* was based on phenotypic analysis and partial 16S rRNA gene sequencing. *Variovorax* sp. strain CSUSB was isolated in an experiment identifying rhizosphere bacteria that grew in the diluted agar medium (0.1% Trypticase soy broth [TSB] solidified with 1.5% agar). Isolates from this medium were subsequently tested for antibiotic resistance. *Variovorax* sp. CSUSB was isolated from the roots of Helianthus annuus, similar to a previous isolation of Variovorax paradoxus EPS (1).

DNA was extracted from cultures of the isolates grown at room temperature in 0.5% yeast extract (Fisher Scientific) broth using the high-molecular-weight DNA protocol outlined for Escherichia coli (https://www.protocols.io/view/ultra-long-read-sequencing-protocol-for-rad004-mrxc57n) (2). These cultures were grown from single colonies picked from plates grown directly from −80°C glycerol stocks, which were made from the initial isolated cultures. Quality assurance and quantitation were performed using the NanoDrop 1 spectrophotometer (Thermo Fisher). Long reads were obtained by preparing libraries using the rapid barcoding kit (catalog number SQK-RBK004) and sequencing these libraries using an MIN-106 flowcell (R9.4.1) in an Oxford Nanopore MinION sequencer. Two separate sequencing runs were performed using barcoded libraries derived from the same genomic sample in separate flow cells, and the data were combined for assembly after processing. Barcodes RB03 and RB06 were used for *Variovorax* sp. CSUSB, and barcodes RB05 and RB04 were used for *P. alkylphenolica* Neo. For all subsequent data-processing steps, the default parameters were used unless otherwise noted. MinION reads were basecalled in Guppy v2.3.1 using the Flipflop model and demultiplexed in Deepbinner v0.2.0 (3). Barcodes and adapters were removed with Porechop v0.2.4 (4). A total of 657,284 reads were obtained for *Variovorax* sp. CSUSB (average read length, 7,561.13 ± 9,721.0 bp; 885.56× coverage), and 35,176 reads were obtained for *P. alkylphenolica* (average read length, 9,573 ± 10,371.88 bp; 60.0× coverage). For *P. alkylphenolica,* the same genomic DNA preparation was used to generate a 250- to 300-bp library with the Nextera DNA Flex LPK kit, which was sequenced in the Illumina iSeq platform (2 × 150 bp). Short read data for *Variovorax* sp. CSUSB were obtained using the FastDNA spin kit for soil (Solon, OH) and sequenced on the Illumina MiSeq platform (500- to 600-bp library fragment size using the Nextera XT kit, 2 × 250 reads). A total of 3,800,551 reads were obtained from the *P. alkylphenolica* library (average read length, 131 bp; 177.43× coverage), and 1,582,102 reads were obtained from the *Variovorax* sp. CSUSB library (average read length, 195.69 ± 40.61 bp; 55.54× coverage). FastQC v0.11.8 was used for quality assessment of these data (5), and trimming was performed in Trimmomatic v0.38.0 (6). Assemblies of *Variovorax* sp. CSUSB and *P. alkylphenolica* Neo were created using a hybrid approach in Unicycler v0.4.8.0 (7) on the North America Galaxy hub (http://usegalaxy.org) (8).

The *Variovorax* sp. CSUSB strain genomic DNA assembled into a single circular contig with 5,574,400 bp. Initial annotation with RASTtk (http://rast.nmpdr.org) (9) identified 5,228 coding sequences and 58 RNAs, with a G+C content of 65.7%. The *P. alkylphenolica* strain assembly generated a single circular contig with 5,612,010 bp, with a G+C content of 61.2%, and a similar annotation identified 5,079 coding sequences and 89 RNAs. The assemblies uploaded to NCBI were annotated using the Prokaryotic Genome Annotation Pipeline (PGAP) by NCBI (10). Using PHASTER (https://phaster.ca/) (11), one complete prophage was identified in the *Variovorax* sp. CSUSB assembly along with two incomplete phage elements, and three complete phage elements were identified in *P. alkylphenolica* Neo.

There is substantial diversity in the genome structure in the genus *Variovorax,* with both single-chromosome and multiple-replicon structures (1, 12). The reported CSUSB strain genome represents one of the smallest *Variovorax* sp. assemblies so far. Variovorax paradoxus NBRC 15149 was identified by 16S rRNA sequence identity as the closest relative (>99%), and the two-way average nucleotide identity (ANI) was calculated (http://enve-omics.ce.gatech.edu/ani/) (13) as 88%. This was the highest level seen in pairwise ANI for this isolate against all sequenced *Variovorax* strains, which leads us to the conservative naming of this isolate as *Variovorax* sp. (14). Analysis of *P. alkylphenolica* Neo revealed high levels of identity by both ANI (two-way ANI, 96.76%) and 16S homology (99.93%) to the sequenced type strain KL28 (15).

### Data availability.

The assemblies and sequence data have been uploaded to the NCBI database. Pseudomonas alkylphenolica Neo can be found at BioProject number PRJNA593854, BioSample number SAMN13494299, and assembly number CP046621. *Variovorax* sp. CSUSB can be found at numbers PRJNA593854, SAMN13494298, and CP046622. Read data can be found at numbers SRR10662237 to SRR10662240, including demultiplexed fastQ files with barcodes removed for the MinION runs and paired fastQ files for the Illumina iSeq and MiSeq runs.
